# Mn^5+^-activated Ca_6_Ba(PO_4_)_4_O near-infrared phosphor and its application in luminescence thermometry

**DOI:** 10.1038/s41377-022-00958-7

**Published:** 2022-09-22

**Authors:** Miroslav D. Dramićanin, Łukasz Marciniak, Sanja Kuzman, Wojciech Piotrowski, Zoran Ristić, Jovana Periša, Ivana Evans, Jelena Mitrić, Vesna Đorđević, Nebojša Romčević, Mikhail G. Brik, Chong-Geng Ma

**Affiliations:** 1 School of Optoelectronic Engineering & CQUPT-BUL Innovation Institute, Chongqing University of Posts and Telecommunications, Chongqing, 400065 PR China; 2 Centre of Excellence for Photoconversion, Vinca Institute of Nuclear Sciences-National Institute of the Republic of Serbia, University of Belgrade, P.O. Box 522, Belgrade, 11001 Serbia; 3 Institute of Low Temperature and Structure Research, Polish Academy of Sciences, Okólna 2, 50-422 Wroclaw, Poland; 4 Department of Chemistry, Durham University, Durham, DH1 3LE UK; 5 Institute of Physics, University of Belgrade, Pregrevica 118, 11080 Belgrade, Serbia; 6 Institute of Physics, University of Tartu, Tartu, 50411 Estonia; 7 Department of Theoretical Physics, Jan Długosz University, Czestochowa PL, 42200 Poland; 8 Academy of Romanian Scientists, Ilfov Str. No. 3, 050044 Bucharest, Romania

**Keywords:** Optical materials and structures, Photonic devices

## Abstract

The near-infrared luminescence of Ca_6_Ba(PO_4_)_4_O:Mn^5+^ is demonstrated and explained. When excited into the broad and strong absorption band that spans the 500–1000 nm spectral range, this phosphor provides an ultranarrow (FWHM = 5 nm) emission centered at 1140 nm that originates from a spin-forbidden ^1^E → ^3^A_2_ transition with a 37.5% internal quantum efficiency and an excited-state lifetime of about 350 μs. We derived the crystal field and Racah parameters and calculated the appropriate Tanabe–Sugano diagram for this phosphor. We found that ^1^E emission quenches due to the thermally-assisted cross-over with the ^3^T_2_ state and that the relatively high Debye temperature of 783 K of Ca_6_Ba(PO_4_)_4_O facilitates efficient emission. Since Ca_6_Ba(PO_4_)_4_O also provides efficient yellow emission of the Eu^2+^ dopant, we calculated and explained its electronic band structure, the partial and total density of states, effective Mulliken charges of all ions, elastic constants, Debye temperature, and vibrational spectra. Finally, we demonstrated the application of phosphor in a luminescence intensity ratio thermometry and obtained a relative sensitivity of 1.92%K^−1^ and a temperature resolution of 0.2 K in the range of physiological temperatures.

## Abstract (web summary)

The Ca_6_Ba(PO_4_)_4_O:Mn^5+^ provides ultranarrow near-infrared emission at 1140 nm that can be excited over 500–1000 nm spectral range, which makes this material an excellent near-infrared phosphor and blue/turquoise pigment.

## Graphical abstract

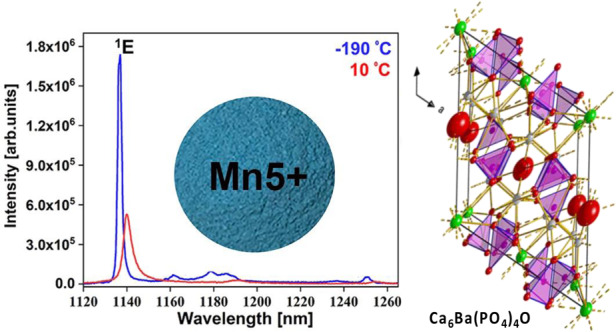

## Introduction

Mn^5+^ optical centers have the [Ar]3*d*^2^ electron configuration and always encounter a strong crystal field when tetrahedrally coordinated in crystals. Their lower electronic states have the ^3^A_2_ < ^1^E < ^1^A_1_ < ^3^T_2_ < ^3^T_1_ progression in energy. The ground state (^3^A_2_) is orbitally non-degenerate and the first excited state ^1^E has almost no nuclear displacement with respect to the ^3^A_2_ state and can be split by the low-symmetry ligand field^1^. The ^1^E energy of approximately 8000–9000 cm^−1^ is strongly affected by a nephelauxetic effect. At low temperatures, emission occurs solely from the spin-forbidden ^1^E → ^3^A_2_ electronic transition of a genuine electric dipole origin. At these temperatures, the emission from the spin-allowed ^3^T_2_ → ^3^A_2_ transition is almost negligible since the low energy orbital of the ^3^T_2_ state is localized more than 1000 cm^−1^ above the ^1^E state, which results in its low population. Therefore, Mn^5+^ emissions appearing in the near infrared (NIR) spectral range at wavelengths longer than 1100 nm and have a narrow spectral band (FWHM < 10 nm) that can be split into two bands with an energy difference of up to 300 cm^−1^. These emission bands are usually accompanied by vibronic sidebands and have decay times of a few tens to a few hundred microseconds.

The ultranarrow-band NIR luminescence of Mn^5+^ is especially favorable for NIR lasers^2–4^ and the development of narrow-band NIR light sources for the selective identification of chemical analytes^5^. Recent research suggests that Mn^5+^ activated nanoparticles are excellent probes for deep-tissue luminescence imaging and luminescence thermometry in the second biological transparency window (1000–1350 nm) and that they show high photo- and chemical stabilities^6,7^. One of their features, especially favorable from the biomedical application perspective, is that they exhibit broad and strong absorption bands from the spin-allowed electronic transitions that span the 500–1000 nm range^8–13^, which facilitates their excitation by the wavelength of the first optical biological window. They have a higher quantum efficiency (QE) than Ag_2_S or Ag_2_Se quantum dots, and do not contain toxic heavy metals like InAs or PbS quantum dots, nor suffer from photobleaching and photoblinking. In comparison to the lanthanide activated NIR bioimaging nanoprobes, Mn^5+^ nanoparticles are brighter due to higher values of the d-d absorption cross-section compared to the spin-forbidden f-f absorptions in the NIR-excited lanthanides. All these advantages of Mn^5+^ indicate that it is an ion with a very important and, so far, unexplored application potential worth large-scale intensive research.

Emissions from Mn^5+^ centers have been demonstrated in a considerably smaller number of host materials compared to Mn^2+^ and Mn^4+^ optical centers^14^. To facilitate Mn^5+^ emission, the material needs to provide both the tetrahedral environment for Mn^5+^ and a sufficiently large energy bandgap compared to the Mn^5+^ transitions’ energies. More importantly, the host material must provide the stabilization of the Mn 5+ valence state, which imposes more constraints on the materials’ structure and composition than the 2+ and 4+ valance states. For these reasons, the majority of Mn^5+^ emitting host materials contain electropositive ions such as alkaline earth metals and PO_4_^3-^ and VO_4_^3-^ groups (or SiO_4_^4−^ group with a charge compensation). The typical examples of such materials are Mn^5+^ activated Li_3_PO_4_^15^, Sr_5_(VO_4_)_3_F^4,16^, Ba_5_(PO_4_)_3_Cl^17^, Sr_5_(PO_4_)_3_Cl^1,17,18^, Ca_2_PO_4_Cl^1,17^, Ca_2_VO_4_Cl^1,17^, Sr_2_VO_4_Cl^1,17^, Y_2_SiO_5_^19^, and M_2_SiO_4_ (M = Ba, Sr, Ca)^20^.

Recently, Kim et al.^21^ have introduced the Ca_6_Ba(PO_4_)_4_O:Mn^5+^ as a new blue pigment that shows coloration due to intense Mn^5+^ absorption, but its luminescent properties have not been analyzed. Also recently, the efficient yellow emission from Eu^2+^ activated Ca_6_Ba(PO_4_)_4_O has been demonstrated^22–24^, implying that Ca_6_Ba(PO_4_)_4_O is an interesting host material for luminescent ions. Considering Ca_6_Ba(PO_4_)_4_O structure and composition, one can observe seven electropositive ions (six Ca and one Ba) surrounding the rigidly connected PO_4_ tetrahedra. Thus, one may assume that the Mn^5+^ emission, in this new host-activator combination, would be efficient and of high energy due to the nephelauxetic effect. For this reason, we prepared the Ca_6_Ba(PO_4_)_4_O:Mn^5+^ powder for this research and observed the intense NIR emission under the 650 nm excitation. The emission energy of 8772 cm^−1^ is among the highest energies detected for Mn^5+^ activated phosphors. Considering the potential use of Ca_6_Ba(PO_4_)_4_O for both yellow and NIR phosphors and the absence of data on its electronic and vibrational properties, we calculated and explained its electronic band structure, the partial and total density of states (DOS), effective Mulliken charges of all constituent ions, elastic constants, Debye temperature, and vibrational spectra of undoped and Mn^5+^ doped material. Then, we proceeded with the detailed characterization and analysis of Ca_6_Ba(PO_4_)_4_O:Mn^5+^ luminescence that includes the measurements of material absorption, excitation and emission spectra, emission decays, concentration quenching, quantum yield, the temperature dependence of emission band shift, bandwidth and decay, and the calculation of the crystal field parameters. This versatile characterization and analysis of Ca_6_Ba(PO_4_)_4_O:Mn^5+^ undoubtedly confirm the high potential of this material in NIR applications.

## Experimental

The conventional solid-state reaction was employed for the preparation of Ca_6_BaP_4-4*x*_Mn_4*x*_O_17_ (*x* = 0.005, 0.0075, 0.0125, 0.01, 0.015, 0.02) powder samples. Stoichiometric amounts of CaCO_3_ (Alfa Aesar, 98%), BaCO_3_ (Alfa Aesar, 99.8%), (NH_4_)H_2_PO_4_ (Alfa Aesar, 98%), and MnO (Aldrich, 99.99%) were thoroughly mixed in an agate mortar for 1 h with an appropriate amount of ethanol. Mixtures of the raw materials were placed in alumina crucibles and heated in an air atmosphere at 600 °C for 6 h, ground in an agate mortar, and further calcinated at 1280 °C for 10 h.

The crystal structure of powders was examined by powder X-ray diffraction (PXRD) using the Rigaku SmartLab instrument (Cu-Kα_1,2_ radiation; λ = 0.1540 nm) at room temperature. Data were recorded over the 6°−130° 2θ range, with a 0.01° step size and 1 min/° counting time. All PXRD data were analyzed by the Rietveld method implemented in TOPAS Academic software^25,26^. Raman scattering measurements were performed using micro – Raman system TriVista 557 equipped with a triple monochromator and CCD detector (monochromator configuration 900/900/1800 points per millimeter) with 1.5 cm^−1^ resolution. For excitation, Ar laser line at 514.5 nm has an incident power of less than 60 mW to minimize the heating effect. Laser beam was focused on the samples by means of microscopic lenses with 100× magnification. Spectra were recorded in the range of 100–1200 cm^−1^. Measurements of diffuse reflectance were performed on the Thermo Evolution 600 spectrometer equipped with an integrated sphere, using BaSO_4_ as a reference over the 220–1350 nm wavelength range. The photoluminescence emission and emission decays were measured using the FLS1000 Fluorescence spectrometer (Edinburgh Instruments; 0.1 nm spectral resolution) supplied with R5509-72 photomultiplier tube from Hamamatsu in nitrogen-flow cooled housing for near-infrared range detection. For measurements of the emission spectra and decays, the 668 nm laser diode excitation is used in the continuous and pulsed mode, respectively. The temperature of the sample was controlled using a THMS 600 heating-cooling stage from Linkam (0.1 K temperature stability and 0.1 K set point resolution). Emission decays at low temperatures were recorded at 1136 nm for all samples. The luminescence quantum efficiencies were measured using FLS980 Fluorescence Spectrometer from Edinburgh Instruments equipped with 450 W Xenon lamp, R5509-72 photomultiplier tube from Hamamatsu in nitrogen-flow cooled housing for near-infrared range detection, and calibrated integrating sphere for the direct absolute efficiency reading. The Al_2_O_3_ powder was used as a scattering reference. Thermometry was performed using a custom-made Peltier-based heating stage in the 20–100 °C range (0.02 °C precision). An Ocean Insight LSM-635A LED was used as the excitation source and is controlled by the Ocean Insight LDC-1 single channel driver and controller. The bifurcation optical Y cable was used for measuring PL emission spectra by Ocean Insight NIRQuest+ Spectrometers.

## Results and discussion

### The structure of Ca_6_Ba(PO_4_)_4_O and Ca_6_Ba(PO_4_)_4_O:Mn^5+^

**Fig. 1:**
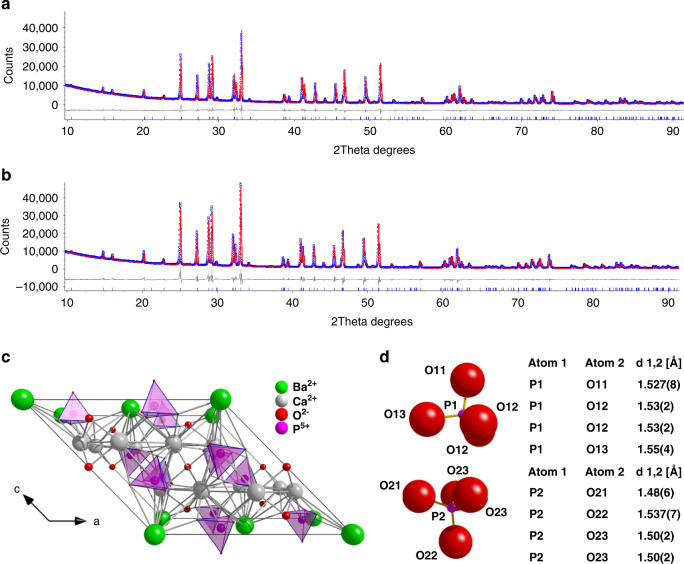
The structure of Ca_6_Ba(PO_4_)_4_O:Mn^5+^. **a** Rietveld fits for Ca_6_Ba(PO_4_)_4_O, R_wp_ = 5.31%. **b** Rietveld fit for Ca_6_Ba(PO_4_)_4_O:Mn^5+^, R_wp_ = 8.59%. In each case the blue curve represents the observed pattern, the red curve is the calculated pattern and the difference curves are shown in grey, while blue tick marks represent the positions of the Bragg peaks. **c** The crystal structure of Ca_6_Ba(PO_4_)_4_O, with the unit cell viewed along the b-axis; **d** Geometries of two PO_4_ tetrahedra with average P–O bond lengths in Ca_6_Ba(PO_4_)_4_O:Mn^5+^

The starting model used for Rietveld refinement and detailed structural analysis of the two key materials – the Ca_6_Ba(PO_4_)_4_O host and the sample containing 0.5%Mn – was the previously published crystal structure of Ca_6_Ba(PO_4_)_4_O determined from synchrotron powder diffraction data^22^. Refined parameters included the zero-point error, background polynomial terms, peak shape function terms, unit cell parameters, an isotropic atomic displacement parameter per atom type and atomic fractional coordinates, using bond valence sum restraints on the two P atoms. The key crystallographic parameters are summarized in Table 1. Ca_6_Ba(PO_4_)_4_O and Ca_6_Ba(PO_4_)_4_O:0.5%Mn adopt monoclinic space group C2/m, with one Ba, two Ca, two P and seven O atoms in the asymmetric unit. Ba atoms are 12-coordinate, the two crystallographically unique Ca atoms are 7- and 8-coordinate, while both unique P atoms adopt tetrahedral coordination environments. Dopant Mn^5+^ ions replace P^5+^ on these two sites, which lie on a mirror plane (Wyckoff site 4 m in space group C2/m). In Ca_6_Ba(PO_4_)_4_O, the average cation-oxygen bond lengths in the two tetrahedra are 1.534(13) and 1.529(15) Å, while bond angles range from 105.1(8) to 112.9(7)° and 105.3(7) to 112.8(9)°, with bond valence sums of 5.0(1) for both P atoms. In Ca_6_Ba(PO_4_)_4_O:0.5%Mn, the coordination environments remain similar, as expected given a low doping level. Average bond lengths are 1.534(22) and 1.504(27) Å, while bond angles range from 103.4(16) to 112.8(12)° and from 105.5(13) to 112.5(11)°, with bond valence sums of 5.0(2) and 5.4(2) for P1 and P2 sites, (see Tables 2 and 3), respectively. The final Rietveld fits obtained are shown in Fig. 1a, b, while the unit cell and the P atom environments are given in Fig. 1c, d, respectively.

**Table 1:** Summary of structural data for Ca_6_Ba(PO_4_)_4_O and Ca_6_Ba(PO_4_)_4_O:Mn^5+^ (space group C2/m)

	Exp. Ca_6_Ba(PO_4_)_4_O			Exp. Ca_6_Ba(PO_4_)_4_O:Mn		
*a*, Å	12.3006 (1)			12.2973 (2)		
*b*, Å	7.10472 (7)			7.10258 (1)		
*c*, Å	11.71540 (9)			11.7125 (1)		
*β*, °	134.4619 (4)			134.4552 (8)		
*V*, Å^3^	730.73 (1)			730.22 (2)		

Fractional atomic coordinates						
	*x*	*y*	*z*	*x*	*y*	*z*
Ba	0	0	0	0	0	0
Ca1	0.7196 (6)	0	0.3117 (8)	0.719 (1)	0	0.311 (1)
Ca2	0.0505 (4)	0.7665 (5)	0.6929 (4)	0.0505 (8)	0.766 (1)	0.6930 (7)
O	0	0	0.5	0	0	0.5
O11	0.399 (1)	0	0.5822 (5)	0.39 4 (2)	0	0.5822 (8)
O12	0.281 (1)	−0.175 (1)	0.3367 (6)	0.281 (2)	−0.175 (2)	0.336 (1)
O13	0.120 (1)	0	0.366 (1)	0.120 (2)	0	0.365 (2)
O21	0.289 (1)	0	0.106 (2)	0.290 (3)	0	0.104 (3)
O22	0.242 (1)	0	−0.1373 (5)	0.241 (3)	0	−0.1368 (7)
O23	0.462 (1)	−0.179 (1)	0.114 (1)	0.461 (2)	−0.174 (2)	0.114 (1)
P1	0.2673 (6)	0	0.3999 (2)	0.266 (1)	0	0.3998 (2)
P2	0.3659 (8)	0	0.0469 (2)	0.365 (1)	0	0.0469 (3)

	Calculated					
	GGA			LDA		
*a*, Å	12.4130			12.0755		
*b*, Å	7.1493			6.9658		
*c*, Å	11.8311			11.4881		
*β*, °	134.3735			134.4363		
*V*, Å^3^	750.489			689.983		

Fractional atomic coordinates						
	*x*	*y*	*z*	*x*	*y*	*z*
Ba	*x*	*y*	*z*	*x*	*y*	*z*
Ca1	0	0	0	0	0	0
Ca2	0.71621	0	0.30969	0.71770	0	0.31126
O	0.05261	0.76649	0.69427	0.05232	0.76734	0.69366
O11	0	0	0.5	0	0	0.5
O12	0.39056	0	0.58173	0.39300	0	0.58450
O13	0.28457	−0.17593	0.34032	0.28484	−0.17824	0.33934
O21	0.11338	0	0.34744	0.11195	0	0.34779
O22	0.29114	0	0.11117	0.29072	0	0.11278
O23	0.24386	0	−0.13571	0.24374	0	-0.13726
P1	0.46648	−0.17433	0.11348	0.46930	−0.17679	0.11594
P2	0.26593	0	0.39888	0.26621	0	0.39890

**Table 2:** P–O bond lengths (Å)

	Undoped Ca_6_Ba(PO_4_)_4_O		Mn^5+^ doped Ca_6_Ba(PO_4_)_4_O	
	P1	P2	P1	P2
	1.530 (5)	1.52 (3)	1.527 (8)	1.48 (6)
	1.523 (13)	1.542 (5)	1.53 (2)	1.537 (7)
	1.523 (13)	1.527 (12)	1.53 (2)	1.50 (2)
	1.56 (2)	1.527 (12)	1.55 (4)	1.50 (2)
Avg:	1.534 (13)	1.529 (15)	1.534 (22)	1.504 (27)

**Table 3:** O**–**P**–**O angles and their difference, δ, to regular tetrahedron angle of 109.5°

Undoped Ca_6_Ba(PO_4_)_4_O				Mn^5+^ doped Ca_6_Ba(PO_4_)_4_O			
P1	δ [°]	P2	δ [°]	Mn1	δ [°]	Mn2	δ [°]
109.8 (12)	0.3	112.8 (9)	3.3	110 (2)	0.5	111.9 (17)	2.4
107.9 (4)	−1.6	105.3 (7)	−4.2	108.8 (8)	−0.7	105.5 (13)	−4.0
107.9 (4)	−1.6	105.3 (7)	−4.2	108.8 (8)	−0.7	105.5 (13)	−4.0
105.1 (8)	−4.4	109.1 (11)	−0.4	103.4 (16)	−6.1	10 9 (2)	−0.5
112.9 (7)	3.4	111.9 (6)	2.4	112.8 (12)	3.3	112.5 (11)	3.0
112.9 (7)	3.4	111.9 (6)	2.4	112.8 (12)	3.3	112.5 (11)	3.0
Avg (δ)	*−0*.*08*		*−0*.*12*	Avg (δ)	*−0*.*07*		*−0*.*42*
Avg (abs(δ))	*2*.*4*		*2*.*8*	Avg (abs(δ))	*2*.*4*		*2*.*8*

### Electronic properties

A detailed calculation of the electronic properties of the Ca_6_Ba(PO_4_)_4_O was performed to verify if its electronic structure is suitable to facilitate the Mn^5+^ emission. The electronic configurations for all chemical elements in Ca_6_Ba(PO_4_)_4_O were as follows: O – 2s^2^2p^4^, P - 3s^2^3p^3^, Ca - 3s^2^3p^6^4s^2^, Ba - 5s^2^5p^6^6s^2^. The following parameters were used for the calculations: energy 10^−5^ eV per atom; maximal force 0.03 eVÅ^−1^; maximal stress 0.05 GPa; maximal displacement 0.001 Å. K-points set was 2 × 2 × 2 for geometry optimization and 3 × 3 × 3 for the DOS calculations; the energy cut-off was 340 eV.

The structures obtained by geometry optimizations using the generalized gradient approximation (GGA) and the local density approximation (LDA) calculations are shown in Table 1 and are in excellent agreement, LDA especially, with the data obtained by Rietveld analysis of PXRD.

**Fig. 2:**
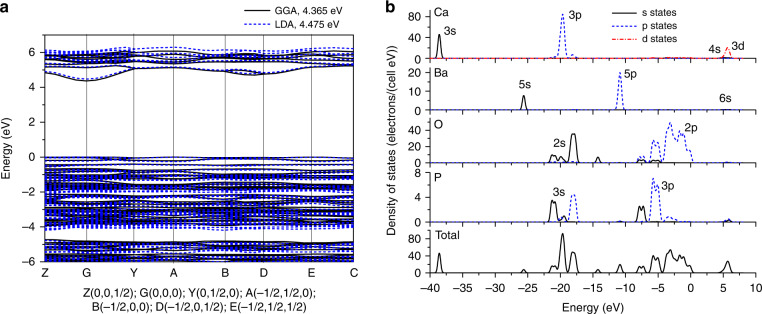
Electronic properties of Ca_6_Ba(PO_4_)_4_O:Mn^5+^. **a** The electronic band structure of Ca_6_Ba(PO_4_)_4_O. **b** Partial and total density of states of Ca_6_Ba(PO_4_)_4_O

The band gaps in both GGA and LDA calculations, Fig. 2a, are direct, equal to 4.365 eV (GGA) and 4.475 eV (LDA) and have similar values to the reported bandgaps of 4.75 eV for the Ca_4_(PO_4_)_2_O and 5.0 eV for the Ba_2_Ca(PO_4_)_2_^27,28^. Since the density-functional theory-based calculations (DFT) always tend to underestimate the true band gaps, the calculated values should be considered as lower band gap estimates.

The conduction band is due to the Ca 4s, 3d and Ba 6s states, Fig. 2b. The top of the valence band is remarkably flat, thus indicating extremely high effective masses of holes. The valence band consists of two sub-bands, which are separated by a narrow gap of about 0.5 eV. The upper one – from about −4.2 eV to 0 eV is made predominantly by the oxygen 2p states. The lower one (from −4.7 eV to about −7 eV) consists of the O 2p states and P 3p states, highly hybridized with each other. The P 3s states make another narrow band between −7.5 eV and −8.5 eV. The O 2s and P 3s states are spread between −22 eV and −17.5 eV, making several clearly seen maxima. The Ba 5s and 5p states are peaked at about −26 eV and −11 eV, respectively, whereas the Ca 3s and 3p states are localized deep in energy at about −38 eV and −20 eV, respectively.

The considered crystal has a high degree of covalency of chemical bonds, which can be assessed by calculating effective Mulliken charges. Since there are two crystallographically inequivalent P^5+^ ions, two Ca^2+^ ions and seven O^2−^ ions and since two types of calculations (GGA/LDA) were run, we give the ranges, in which these charges fall for all ions. They are −1.07-1.09 (in units of proton charge) for the oxygen ions, +2.25 + 2.34 for the phosphorus ions, +1.25 + 1.31 for the calcium ions, and +1.39 + 1.47 for the barium ions. The deviation from the formal charges is especially large for the P and O ions, whereas the Ca and Ba effective charges are closer to their formal ones. This is consistent with the P–O bonds being more covalent than the Ca–O and Ba–O ones.

With the calculated elastic constants, it is possible to estimate the Debye temperature using the following equations:

1 $$\theta _D = \frac{h}{k}\left( {\frac{{3n}}{{4\pi }}\frac{{N_A\rho }}{M}} \right)^{1/3}v_m$$

2 $$v_m = \left[ {\frac{1}{3}\left( {\frac{2}{{v_t^3}} + \frac{1}{{v_l^3}}} \right)} \right]^{^{ - 1/3}}$$

3 $$v_l = \sqrt {\frac{{3B + 4G}}{{3\rho }}} ,\;v_t = \sqrt {\frac{G}{\rho }}$$

where *h* = 6.626 × 10^−34^ J·s is the Planck’s constant; *k*_*B*_ = 1.381 × 10^−23^ JK^−1^ is the Boltzmann constant; *N*_*A*_ = 6.022 × 10^23^ mol^−1^ is the Avogadro’s number; *ρ* is the crystal’s density; *n* is the number of atoms per one formula unit (twenty eight in the case of Ca_6_Ba(PO_4_)_4_O), and *M* is the formula weight. The average, transverse and longitudinal sound velocities are denoted by *v*_*m*_, *v*_*t*_, *v*_*l*_, correspondingly. The B (bulk modulus) and G (shear modulus) values are calculated as the average values of the corresponding Voigt and Reuss (denoted with the V and R subscripts, respectively) values from Table 4. With these equations and calculated elastic parameters, the Debye temperature for Ca_6_Ba(PO_4_)_4_O was estimated to be 496 K (GGA) and 551 K (LDA).

**Table 4:** Calculated elastic constants (in GPa) for Ca_6_Ba(PO_4_)_4_O

	GGA	LDA
*C*_11_	127.8	162.1
*C*_22_	141.2	180.3
*C*_33_	149.1	186.3
*C*_44_	29.7	42.4
*C*_55_	48.0	60.7
*C*_66_	55.5	67.3
*C*_12_	62.7	81.8
*C*_13_	45.5	62.8
*C*_15_	4.0	−2.0
*C*_23_	38.9	56.4
*C*_25_	−4.7	−11.7
*C*_35_	−5.2	−7.7
*C*_46_	3.5	−3.8
*B*_*V*_	79.2	103.4
*B*_*R*_	79.0	102.5
*G*_*V*_	44.7	55.9
*G*_*R*_	41.6	53.3

### Vibrational spectra

**Fig. 3:**
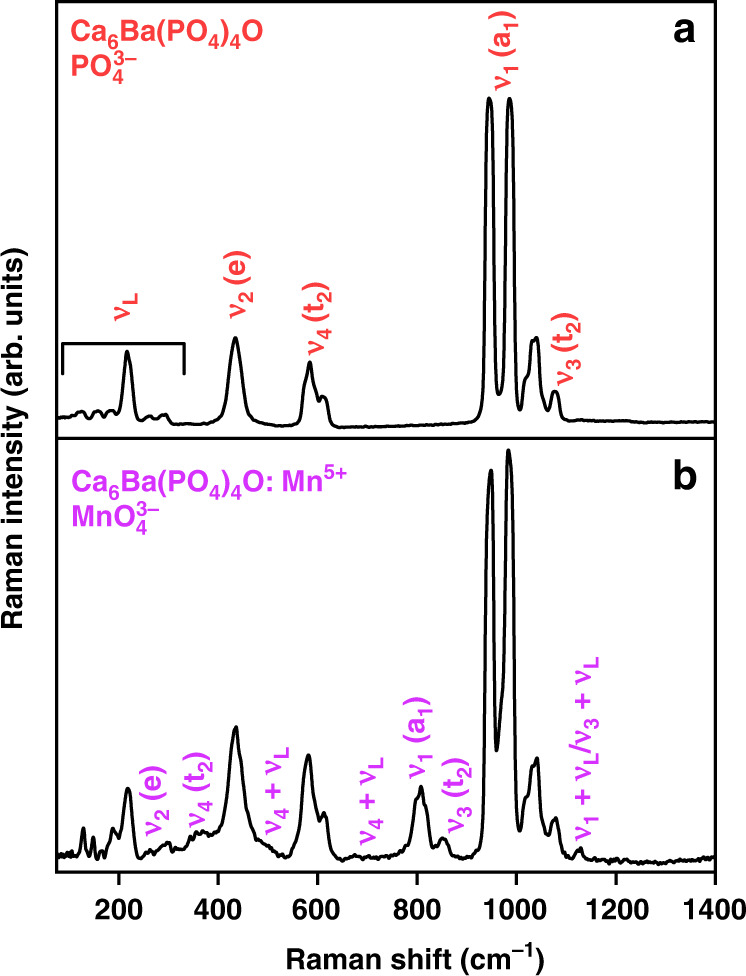
Vibrational properties of Ca_6_Ba(PO_4_)_4_O:Mn^5+^. **a** Raman scattering spectra of Ca_6_Ba(PO_4_)_4_O powder. **b** Raman scattering spectra of Ca_6_Ba(PO_4_)_4_O:Mn^5+^ powder

For an undistorted tetrahedron (T_d_ symmetry), the four fundamental vibrational modes (all Raman active) are a_1_ + e + 2t_2_^1,29,30^. The ν_1_(a_1_) is the totally symmetric stretching mode, ν_2_(e) is bending deformation, and ν_3_(t_2_) and ν_4_(t_2_) vibrations are the two stretching t_2_ modes. With symmetry lowered from T_d_ to C_s_, several bands appear for each vibrational mode. Raman scattering spectra of the Ca_6_Ba(PO_4_)_4_O and Ca_6_Ba(PO_4_)_4_O:Mn^5+^ powders are shown in Fig. 3a, b, respectively. The following assignment is made for the Ca_6_Ba(PO_4_)_4_O: ν_L_ (cm^−1^) = 123, 153, 183, 215.4, 262.5, and 294.7 are the lattice modes; ν_1_(a_1_) (cm^−1^) = 944 and 985.3; ν_2_(e) = 434.5 (cm^−1^); ν_3_(t_2_) (cm^−1^) = 1040.1, and 1074.2; ν_4_(t_2_) (cm^−1^) = 584.3 and 608.3. In Raman scattering spectra of the Ca_6_Ba(PO_4_)_4_O:Mn^5+^ powder, Fig. 3b, additional vibrations from the MnO_4_^3−^ ion are clearly visible: ν_1_(a_1_) (cm^−1^) = 807; ν_2_(e) (cm^−1^) = 314.4; ν_3_(t_2_) (cm^−1^) = 851.4; ν_4_(t_2_) (cm^−1^) = 343.9, 356.2, and 368.5; ν_1+_ ν_L_/ ν_3+_ ν_L_ (cm^−1^) = 1128.5 and >1128.5. They agree with the data reported by Gonzalez-Vilchez and Griffith^29^ for vibrational modes of the MnO_4_^3−^ molecular ion.

### Photoluminescence properties

**Fig. 4:**
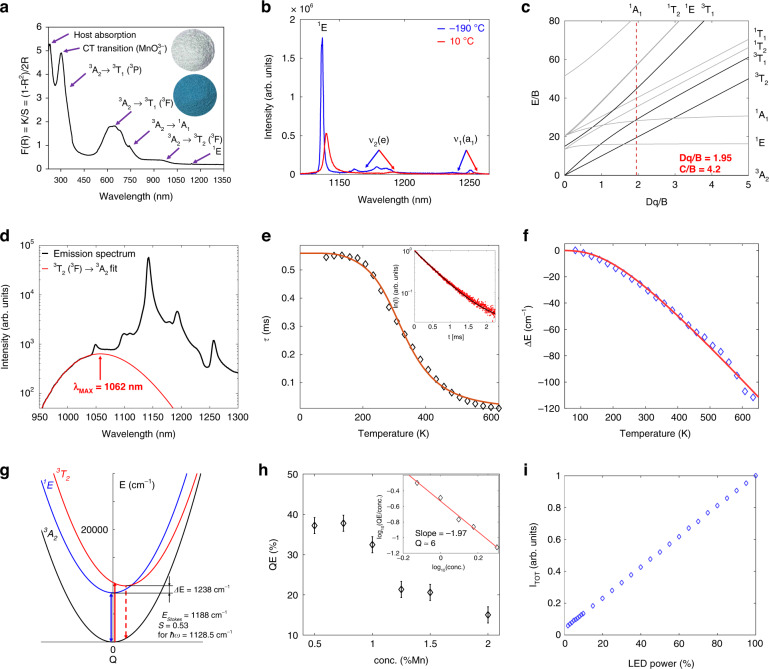
Optical properties of the Ca_6_Ba(PO_4_)_4_O:Mn^5+^ powder. **a** The Kubelka−Munk transformation of the Ca_6_Ba(PO_4_)_4_O:Mn^5+^ diffuse reflectance The inset shows photographs of the Ca_6_Ba(PO_4_)_4_O (white) and Ca_6_Ba(PO_4_)_4_O:Mn^5+^ (blue) powders. **b** Emission spectra measured at −190 °C and 10 °C. **c** Tanabe–Sugano diagram for 3d^2^ electron configuration in tetrahedral coordination. **d** Emission spectrum of the Ca_6_Ba(PO_4_)_4_O:Mn^5+^ measured at room temperature (black line) and the fit to the Gaussian of the ^3^T_2_ →^3^A_2_ emission peak (red line) showing its maximum 1062 nm/9416 cm^−1^. Spectra shown in logarithmic scale. **e** Temperature dependence of the excited state lifetime (symbols – experimental data, solid line – the fit to Eq. (4)). The inset shows emission decay measured at 208 K. **f** Temperature dependence of the ^1^E emission peak spectral position (symbols – experimental data, solid line – the fit to Eq. (5)). **g** The estimate of configurational diagram based on the spectroscopic data with calculated Stokes shift (E_Stokes_) and Huang–Rhys parameter (S). **h** Photoluminescence internal quantum efficiency (QE) of Ca_6_Ba(PO_4_)_4_O:Mn^5+^ powders for different concentrations of Mn. The inset shows linear dependence of the log_10_(QE/concentration) vs log_10_(concentration) for data equal and above critical concentration (0.75%) with a slope of −1.97 indicating that a multipolar dipole-dipole mechanism is responsible for the concentration quenching of emission. **i** The linear dependence of Ca_6_Ba(PO_4_)_4_O:Mn^5+^ emission intensity on excitation power

Figure 4a, b displays the absorption, color (inset in Fig. 4a), and emission spectra of Ca_6_Ba(PO_4_)_4_O:0.5%Mn^5+^ powder. The observed absorption and emission, the blue coloration of the Mn^5+^ doped powder, and the characteristic vibrational modes of MnO_4_^3−^ in the Raman scattering spectrum of Ca_6_Ba(PO_4_)_4_O:Mn^5+^ (Fig. 3b) all unambiguously demonstrate the presence of Mn^5+^ in the sample.

Figure 4a depicts the Kubelka–Munk transformation of the Ca_6_Ba(PO_4_)_4_O:Mn^5+^ powder diffuse reflection measured between 220 and 1350 nm. The O^2−^ → Mn^5+^ charge-transfer band appears at around 301 nm (33,222 cm^−1^) as expected for the tetraoxo-coordinated Mn^5+^ ^31^ and the peak at the lower wavelength (225 nm) is associated with the intrinsic host absorption. The strong absorption around 639 nm (15,649.5 cm^−1^) is associated with the ^3^A_2_ → ^3^T_1_(^3^F) electronic transition, which is electric dipole-allowed in an undistorted tetrahedral symmetry and is composed of three overlapping components due to the removal of the orbital degeneracy of the ^3^T_1_(^3^F) state with the site symmetry lowering from *T*_*d*_ to *C*_*s*_. The weak shoulder at about 943 nm (10,604.5 cm^−1^) corresponds to the symmetry forbidden ^3^A_2_ → ^3^T_2_(^3^F) transition (in *T*_*d*_ site symmetry) and becomes partially allowed with a symmetry lowering. The electric dipole-allowed ^3^A_2_ → ^3^T_1_(^3^P) transition that corresponds to a two-electron jump is located at approximately 369 nm (27,100 cm^−1^) and is barely visible due to the much more intense charge transfer band. The spin-forbidden transitions to the singlet states ^1^A_1_(^1^G) at 740 nm (13,513.5 cm^−1^) and ^1^E(^1^D) at 1140 nm (8772 cm^−1^) are weak, sharp, and only weakly depend on the host materials properties. The transitions to ^1^T_1,2_ singlet states are difficult to observe in the spectrum since they are very weak and superimposed on the main and stronger bands.

Emission spectra of Ca_6_Ba(PO_4_)_4_O:Mn^5+^ powder measured at −190 °C and 10 °C are shown in Fig. 4b with blue and red lines, respectively, and are typical for emissions from transitions of 3d^2^ electronic configuration in a tetrahedral environment as described by the Tanabe–Sugano diagram, Fig. 4c. The spectra show ultranarrow emission bands (FWHM = 3 nm (20 cm^−1^) at −190 °C; FWHM = 5 nm (35 cm^−1^) at 10 °C) from the ^1^E → ^3^A_2_ intraconfigurational transition (1140 nm), followed by vibrational sidebands with progressions of ≈320 cm^−1^ ((ν_2_(e)) and ≈800 cm^−1^ ((ν_1_(a_1_)). This indicates the coupling of the ^1^E excited state and the non-totally symmetric ν_2_(e) mode of MnO_4_^3−^, i.e., a dynamic Jahn–Teller effect.

The very small splitting of ^1^E emission band is due to only weakly distorted MnO_4_ tetrahedra (see Table 3) and it is barely visible with our instrument resolution at the emission spectrum measured at low temperatures (−190 °C). The low-intensity broad emission band from the ^3^T_2_(^3^F) → ^3^A_2_ transition is centered at 1062 nm (9416 cm^−1^) and can be resolved only spectral deconvolution, Fig. 4d.

Temperature dependence of the ^1^E lifetime and emission peak spectral position are shown in Fig. 4e, f, respectively. The ^1^E level emission decays show lifetime values of about 350 μs at room temperature and 560 μs at low temperatures. The temperature dependence of lifetime, Fig. 4e, shows that a low-temperature lifetime value is approximately the value of a radiative lifetime. Considering that the excited state is separated in energy from the ground state by 8772 cm^−1^, almost eight quanta of the highest vibrational frequencies of the phosphor (≈1100 cm^−1^) are needed to bridge the gap. Thus, a multiphonon non-radiative relaxation is not probable as the emission quenching mechanism. The ^1^E emission deactivation through the crossing with a charge transfer band is also not probable due to very high energy difference. Therefore, we assume that the thermal quenching of the ^1^E state population takes place by a thermally activated cross-over via ^3^T_2_ state, see Fig. 4g, similarly to Mn^4+^ activated phosphors. The temperature dependence of the emission lifetime, shown in Fig. 4e, can be described by the following equation^32–35^:

4 $$\tau \left( T \right) = \frac{{\tau _{R0} \cdot {{{\mathrm{tanh}}}}(h\nu /2k_BT)}}{{1 + \left( {\tau _{R0} \cdot {{{\mathrm{tanh}}}}(h\nu /2k_BT)/\tau _{NR}} \right) \cdot \exp \left( { - \Delta E/k_BT} \right)}}$$

where $\tau _{R0}$ = 560 ± 19 μs is the radiative lifetime at *T* = 0 K, *k*_*B*_ = 0.69503476 cm^−1^K^−1^ is the Boltzmann constant, $h\nu$ = 448 ± 90 cm^−1^ is the average energy of phonon coupled to the ^1^E → ^3^A_2_ transition, 1/$\tau _{NR}$ = 1527 ± 120 ms^−1^ is the nonradiative decay rate, $\Delta E$ = 1631 ± 200 cm^−1^ is the activation energy of the process (the cross-over via the ^3^T_2_ state), and *T* represents the temperature. The smaller the configuration coordinate parabola offset between the ground state (^3^A_2_) and the ^3^T_2_ state, the larger the cross-over energy $\Delta E$ (activation energy of the process) needed to activate the non-radiative de-excitation process. Thus, the ^1^E → ^3^A_2_ emission of Mn^5+^ activated phosphors, which have large ^3^T_2_ energies and smaller Stokes shifts, will start to quench at higher temperatures.

The shift of ^1^E emission band with energy is shown in Fig. 4f. It can be described by the following equation:^36,37^

5 $$\delta E\left[ {{{{\mathrm{cm}}}}^{ - 1}} \right] = \alpha \cdot \left( {\frac{T}{{\theta _D}}} \right)^4 \cdot {\int}_0^{T/\theta _D} {\frac{{x^3}}{{e^x - 1}}dx}$$

where $\theta _D$ = 783 ± 12 K is the Debye temperature of the host material, $x = \hbar \omega _D/k_BT = \theta _D/T$, $\omega _D$ is Debye cut-off frequency, and *α* = −650 ± 17 cm^−1^ represents the electron-phonon coupling coefficient. The relatively high Debye temperature indicates a rigid structure which favors efficient emissions from optical centers^38^.

The concentration dependence of an internal quantum efficiency (QE) is given in Fig. 4h. The largest value of 37.5 ± 2.0% is recorded for the 0.5% Mn^5+^ doped sample, after which the concentration quenching of emission occurs. This is a relatively high value for an NIR-emitting phosphor, and comparable to one obtained in Ba_3_(PO_4_)_2_^7^. The log_10_(QE/concentration) vs log_10_(concentration) plot has a −1.97 slope, which is close to −2, which undoubtedly indicates that a multipolar electric dipole-dipole mechanism is responsible for the concentration quenching of emission. The linear dependence of Ca_6_Ba(PO_4_)_4_O:Mn^5+^ emission intensity on excitation power, Fig. 4i, is expected for the typical downshifting photoluminescence emission process.

The 3d^2^ electronic configuration of Mn^5+^ in a tetrahedral environment is described by the Tanabe–Sugano model for 3d^8^ electronic configuration in octahedral symmetry^39^, see Fig. 4c. The crystal field and Racah parameters are calculated from the following equations using data from diffuse reflection and emission spectra^40,41^:

6 $$Dq = \frac{{E\left( {{}^3A_2 \to {}^3T_2} \right)}}{{10}} = \frac{{10604.5}}{{10}}\;{{{\mathrm{cm}}}}^{ - 1} = 1060\;{{{\mathrm{cm}}}}^{ - 1}$$

7 $$\begin{array}{l}x = \displaystyle\frac{{E\left( {{\,}^3A_2 \to {\,}^3T_1} \right) - E\left( {{\,}^3A_2 \to {\,}^3T_2} \right)}}{{Dq}}\\\quad =\, \displaystyle\frac{{15649.5 - 10604.5}}{{1060.45}} = 4.757\end{array}$$

8 $$B = \frac{{x^2 - 10x}}{{15 \cdot (x - 8)}} \cdot Dq = 544\;{{{\mathrm{cm}}}}^{ - 1} \to \frac{{10Dq}}{B} = 19.5$$

9 $$\begin{array}{l}C = \displaystyle\frac{1}{2} \cdot \left( {E\left( {{\,}^3A_2 \to {\,}^1E} \right) - 10Dq - 8.5B + \frac{1}{2}\sqrt {400Dq^2 + 40DqB + 49B^2} } \right)\\\quad =\, 2292\;{{{\mathrm{cm}}}}^{ - 1}\end{array}$$

10 $$C/B = 4.21$$

By comparing the obtained *Dq*, *B* and *C* parameters with literature data, Table 5, one can observe that Ca_6_Ba(PO_4_)_4_O provides the smallest *Dq* and the largest *B* parameters amongst all phosphate hosts, and that Li_3_VO_4_ is the only host with a smaller *Dq* (considering available data).

**Table 5:** Comparison of the *Dq*, *B* and *C* parameters (all in cm^−1^) for the tetrahedrally coordinated Mn^5+^ ions in different crystalline solids

Host material	*Dq*	*B*	*C*	Reference
Li_3_PO_4_	1208	475	2556	^49^
Ca_2_PO_4_Cl	1162	455	2657	^49^
Y_2_SiO_5_	1133	550	2255	^50^
Sr_5_(PO_4_)_3_Cl	1100	500	2320	^3^
YAlO_3_	1100	485	2256	^51^
Sr_10_(VO_4_)_6_F_2_	1088	518	2321	^52^
Ca_6_Ba(PO_4_)_4_O	1060	544	2292	*This work*
Li_3_VO_4_	1049	646	2006	^53^

By considering the obtained parameters and the configuration coordinate diagram, Fig. 4g, the relatively small value of Huang–Rhys parameter S = 0.53 is found for the assumed coupling to the ν_1+_ ν_L_/ ν_3+_ ν_L_ vibrational mode with energy $\hbar \omega = 1128.5\;{{{\mathrm{cm}}}}^{ - 1}$.

The Slater parameters are calculated from Racah parameters by the simple relations^42,43^:

11 $$F^{\left( 2 \right)} = 49F_2 = 7\left( {7B + C} \right) = 42271{{{\mathrm{cm}}}}^{ - 1}$$

12 $$F^{(4)} = 441F_4 = 441\frac{C}{{35}} = 28877\;{{{\mathrm{cm}}}}^{ - 1}$$

Both values are considerably reduced from the free-ion values of $F^{\left( 2 \right)} = 91427{{{\mathrm{cm}}}}^{ - 1}$ and $F^{(4)} = 56625\;{{{\mathrm{cm}}}}^{ - 1}$^18^.

As it follows from the Tanabe–Sugano diagram for the 3d^2^ configuration in the tetrahedral crystal field (Fig. 4c), the energy separation between the ground state ^3^A_2_ and the first excited state ^1^E (in the strong crystal field) is practically independent on the crystal field strength (both states are parallel to each other). At the same time, this energy interval is very close to the energy interval between the ^3^F and ^1^D states of the free ion, which is determined by the Racah parameters *B* and *C*, which vary from host to host because of the covalent effects. As a result, the nephelauxetic effect is dominating in this case.

### Application in luminescence thermometry

**Fig. 5:**
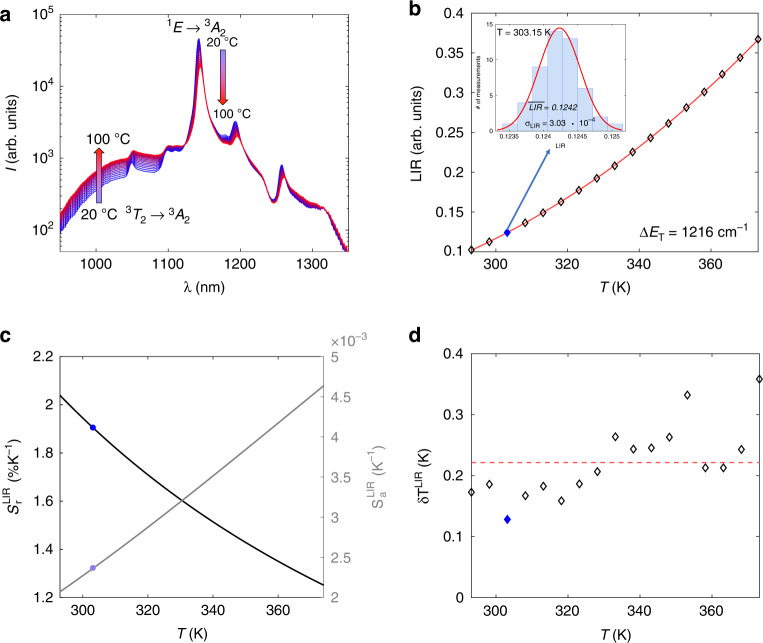
Luminescence thermometry with Ca_6_Ba(PO_4_)_4_O:Mn^5+^ powder. **a** Photoluminescence emission spectra of Ca_6_Ba(PO_4_)_4_O:Mn^5+^ powder measured at different temperatures. **b** Luminescence intensity ratio (LIR) as a function of temperature (experimental data - diamond markers; fit to Eq. (13) - full line). The insert shows the LIR distribution histogram measured at 303.15 K (30 °C) – filled diamond marker. **c** Calculated absolute and relative sensitivities (marked values at 303.15 K (30 °C)). **d** Experimentally obtained values for temperature resolution – filled diamond marker represents the value at 303.15 K (30 °C)

We have tested the performance of Mn^5+^ activated Ca_6_Ba(PO_4_)_4_O (the sample containing 0. 5% Mn since it showed the best quantum efficiency) as a NIR luminescent thermometer operating in the second biological window and in the physiological temperature range. As can be seen from Fig. 5a, when temperature increases, the broad emission peak from the ^3^T_2_ level in the 950 nm to 1030 nm range also increases in intensity, while the intensity of the narrow emission peak from the ^1^E level around 1140 nm decreases with temperature. This occurs due to thermalization between ^1^E and ^3^T_2_ levels where the energy difference between these two levels ($\Delta E_T$) is bridged by thermally exited electrons. Consequently, a simple Boltzmann-type relation for the luminescence intensity ratio (LIR) between the two abovementioned emission intensities applies^44,45^:

13 $$LIR\left( T \right) = \frac{{I({\,}^3T_2)}}{{I({\,}^1E)}} = B \cdot \exp \left( { - \frac{{\Delta E_T}}{{k_BT}}} \right)$$

where *B* is a temperature-invariant constant and *T* represents temperature. The fit of Eq. (13) (full line, Fig. 5b) to experimental LIR data (diamond markers, Fig. 5b) is almost perfect (R^2^ = 0.997) and provides an energy difference $\Delta E_T\;{{{\mathrm{of}}}}\;1216\;{{{\mathrm{cm}}}}^{ - 1}$ that agrees with the energy difference obtained from spectroscopy (Fig. 4g). To experimentally determine the uncertainty in the LIR (error budget), 50 emission spectra were acquired at each temperature. Then, the measurement distribution mean was used as the LIR value while a standard deviation ($\sigma _{LIR}$) was used as an uncertainty in LIR as shown in the insert of Fig. 5b on the LIR value distribution measured at 30 °C).

The absolute (S_a_) and relative (S_r_) sensitivities of the thermometer were then calculated from the following equations:

14 $$S_a\left[ {{{{\mathrm{K}}}}^{ - 1}} \right] = \left| {\frac{{\partial LIR}}{{\partial T}}} \right|,\;S_r\left[ {{{{\mathrm{\% K}}}}^{ - 1}} \right] = 100{{{\mathrm{\% }}}} \cdot \left| {\frac{{\partial LIR}}{{\partial T}}\frac{1}{{LIR}}} \right|$$

and presented in Fig. 5c (blue dots represent values obtained at a temperature of 30 °C). The relative sensitivity value varies from 2.35%K^−1^ to 1.26%K^−1^ over the measurement range, being 1.92%K^−1^ at 30 °C. These are relatively high values^46^, especially for luminescence thermometers operating in the second biological transparency window (>1000 nm). For example, Gschwend et al.^47^ achieved a relative sensitivity of 0.43%K^−1^ for an LIR thermometer based on Mn^5+^-activated Ba_3_(PO_4_)_2_, while Shen et al.^48^ achieved a relative sensitivity of 1.3%K^−1^ for an LIR thermometer based on Ag_2_S quantum dots.

The temperature resolution (uncertainty in measured temperature, $\delta T$) is determined as a ratio between experimentally obtained LIR uncertainty ($\sigma _{LIR}$) and absolute sensitivity ($S_a$) for a given temperature, Fig. 5d:

15 $$\delta T = \frac{{\sigma _{LIR}(T)}}{{S_a(T)}}$$

and it has an average value of 0.21 K. Finally, repeatability of measurement (*R*_*M*_) is quantified as^46^:

16 $$R_M = 1 - \frac{{max\left| {\overline {LIR} - LIR_i} \right|}}{{\overline {LIR} }}$$

where $\overline {LIR}$ is the average LIR measured at a certain temperature over all $LIR_i$ acquired. Based on experimental data, an *R*_*M*_ value of 0.97 (97%) is obtained.

## Conclusion

Because of its rigid structure, appropriate crystal sites for doping, and sufficiently large energy band gap to accommodate the energy levels of dopant ions, Ca_6_Ba(PO_4_)_4_O is an excellent host for Eu^2+^, Sm^2+^ and Mn^5+^ luminescence centers. In this host, Mn^5+^ provides ultranarrow emission in the near-infrared spectral range at 1140 nm that can be easily excited over the broad spectral range that spans 500–1000 nm covering the entirety of the first biological transparency window and making this material an excellent near-infrared phosphor and non-toxic blue/turquoise pigment. The phosphor has an internal quantum efficiency of 37.5%, which is a high value considering the quantum efficiencies of inorganic NIR phosphors. The thermal quenching of the ^1^E emission takes place by a thermally activated cross-over via the ^3^T_2_ state with an activation energy of 1631 cm^−1^. The optimal Mn^5+^ doping concentration is 0.5%. For higher doping concentrations, quantum efficiency decreases due to non-radiative deexcitation caused by a dipole-dipole electric interaction. Based on the available literature data, the Ca_6_Ba(PO_4_)_4_O:Mn^5+^ phosphor provides the smallest *Dq* and the largest *B* parameters amongst all phosphate hosts. This material is one of the best single-doped ratiometric luminescence thermometry sensors for use in a second biological transparency window due to the opposite temperature dependence of the ^1^E and ^3^T_2_ emission intensities. It provides a relative sensitivity of 1.92%K^−1^ and a temperature resolution of 0.2 K in the range of physiological temperatures, with a measurement repeatability of 97%. These findings, particularly the high value of quantum efficiency and strong absorption, tuneability of emission wavelength by changing the Mn^5+^ environment, long emission decay times that allow for time-gated measurements, and strong temperature susceptibility of emission, demonstrate the great potential of Mn^5+^-activated phosphors for NIR applications. When reduced to nano dimension, Ca_6_Ba(PO_4_)_4_O:Mn^5+^ has great potential for bioimaging and biothermal imaging applications in the second biological transparency window. Future research will concentrate on the synthesis and applications of Ca_6_Ba(PO_4_)_4_O:Mn^5+^ nanoparticles.

## Data Availability

The data that support the findings of this study are available from the corresponding author upon reasonable request.
